# Oral consumption of α-glucosyl-hesperidin could prevent lens hardening, which causes presbyopia

**DOI:** 10.1016/j.bbrep.2020.100885

**Published:** 2020-12-26

**Authors:** Yosuke Nakazawa, Miki Aoki, Yuri Doki, Naoki Morishita, Shin Endo, Noriaki Nagai, Megumi Funakoshi-Tago, Hiroomi Tamura

**Affiliations:** a Faculty of Pharmacy, Keio University, Tokyo, Japan; b R&D Division, Hayashibara Co., Ltd, Okayama, Japan; c Faculty of Pharmacy, Kindai University, Osaka, Japan

**Keywords:** α-glucosyl hesperidin, Hesperetin, Anti-Presbyopia effects, Sclerosis of the lens, Anti-oxidants, Hst: Hesperetin: Hsd: Hesperidin: G-Hsd: α-glucosyl hesperidin: GSH, Reduced glutathione: AsA, Ascorbic acid: ROS, Reactive Oxygen Species: CAT, Catalase: SOD, Superoxide Dismutase

## Abstract

Presbyopia is one of the most well-known diseases of the eye, predominantly affecting the adult population after 50 years’. Due to hardening of the lens and failure of accommodative change, patients lose the ability to focus on near objects. This eye symptom is reported to be an early symptom of age-related nuclear cataract, and we have previously reported that hesperetin treatment could delay the onset of nuclear cataractogenesis induced by sodium selenite. In this study, we examined whether oral intake of α-glucosyl-hesperidin (G-Hsd), which has greater water solubility than hesperetin, could delay the onset of presbyopia. G-Hsd treatment protected lens elasticity, upregulated the mRNA expression of anti-oxidative enzymes like glutathione reductase and thioredoxin reductase 1 in the plasma and lens, and prevented premature cataract symptoms in selenite-induced cataract rat lens. Thus, the anti-presbyopic effects of G-Hsd were attributed, at least in part, to its antioxidant effects. G-Hsd represents the first oral treatment agent with anti-presbyopia and/or anti-cataract properties.

## Highlights

• G-Hsd prevents presbyopia by inhibiting aging-induced lens hardening.
• It reduces aging-induced oxidative stress in the lens and plasma.
• It ameliorates cataract development by preventing sclerosis of the eye.
• G-Hsd is the first oral supplement with anti-cataract and anti-presbyopia effects.

## Graphical abstract

**Figure:**
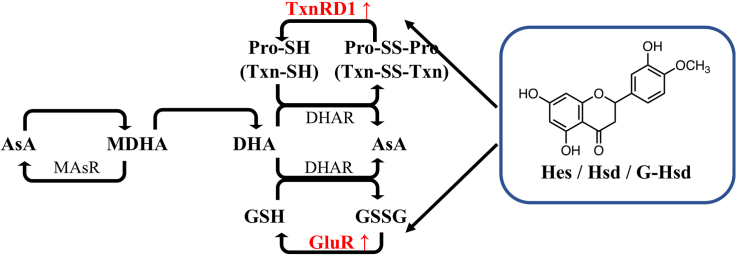

## 1 Introduction

The lens is an important transparent avascular organ that functions to transmit light from the outside and focus an image on the retina. Presbyopia is an eye disease that affects almost 100% of the adult population in their 50s. This disease is characterised by a profound increase in stiffness of the lens resulting in failure to provide accommodative change and an inability to focus on near objects. Currently, there is no cure for presbyopia, but symptoms can be relieved by the use of presbyopia glasses. Aging is associated with phenotypic changes in the lens, ciliary muscles, and zonule fibres. Koopmants et al. reported that sclerosis of the lens is an important inducer of presbyopia and demonstrated that soft-polymer refilling of the presbyopic lens restored the ability to provide accommodative change and enabled focusing on near objects [1]. Presbyopia is an early symptom of age-related nuclear cataract [2]. Age-related changes in the redox state of the lens, including a decrease in reduced glutathione (GSH) and ascorbic acid (AsA) levels, may promote the onset of presbyopia and nuclear cataracts [3]. Therefore, daily intake of antioxidants is a useful approach for the prevention of both presbyopia and cataract formation. In this study, we investigated the effects of hesperetin (Hst), a natural antioxidant, which we have previously reported to have anti-cataract effects in a selenite-induced cataract rat model [4,5]. Hst is the aglycone of hesperidin (Hsd), which is the major flavonoid in citrus fruits. Hsd is composed of an aglycone Hst and rutinose (glucose and rhamnose). However, Hst and Hsd are extremely difficult to dissolve in water, and oral bioavailability is limited. On the contrary, α-glucosyl Hsd (G-Hsd), prepared by Yamada et al., has a solubility in water approximately 10,000 times greater than that of Hst and Hsd, with more than three-fold increased bioavailability [6]. We previously reported that oral intake of G-Hsd ameliorated cataract formation in a rat model [5]. However, no studies have investigated of the effects of Hst and/or G-Hsd in preventing the onset of presbyopia. Therefore, in the current study, we investigated whether oral consumption of G-Hsd oral can prevent age-related changes in the lens and inhibit the formation of immature cataracts in a selenite-induced rodent cataract model.

## 2 Materials and methods

### 2.1 Materials

G-Hsd (including > 80% α-glucosyl Hsd) was provided by Hayashibara Co. (Okayama, Japan). C57Black/6 (C57BL/6) mice and Sprague–Dawley (SD) rats were obtained from Japan SLC Inc. (Shizuoka, Japan). Nutrition-balanced chow for animals (CE-2) was obtained from Clea Japan Inc. (Tokyo, Japan). Dithionitrobenzene (DTNB), trichloroacetic acid, and penicillin-streptomycin antibiotic mixture were purchased from Nakalai Tesque Inc. (Kyoto Japan). Isoflurane, sodium selenite, GSH, AsA, 2,6-Dichlorophenolindophenol (DCPIP) and metaphosphoric acid were purchased from Wako Pure Chemical Industries, Ltd. (Osaka, Japan). The CAT assay kit for catalase (CAT) activity was obtained from Cayman Chemical Inc (Ann Arbor, MI). The GSSG/GSH Quantification kit and SOD assay kit-WST was purchased from Dojindo molecular Technologies, Inc. (Kumamoto, Japan). GlutaMAX and Dulbecco's Modified Eagle Medium/Nutrient Mixture F-12 (DMEM/F-12) were obtained from Gibco-Thermo Fisher Scientific Inc. (Waltham, MA).

### 2.2 Animals

Animals used in this study were housed in a temperature-controlled (23 °C ± 5 °C) environment on a 12 h regular light/dark cycle. Mice and rats were sacrificed with an overdose of isoflurane inhalation. The Keio University Animal Research Committee approved all animal experiments performed in this study (12048-(4)). All animals were treated according to the National Institutes of Health guidelines for the care and use of laboratory animals.

### 2.3 G-Hsd treatment for animals

9-week-old of eighteen C57BL/6J mice were separated randomly into three groups and were given either water (0% G-Hsd group: control), 1% G-Hsd-containing water (1% G-Hsd group), or 2% G-Hsd-containing water (2% G-Hsd group) for 28 weeks. All mice had unrestricted access to chow and drinking water. Every three days, volumes of food and drinking water intake and body weight were measured. Drinking water intake (mL) was calculated by multiplying weight by specific gravity (specific gravity of 1% G-Hsd or 2% G-Hsd solution is 0.997 g/mL or 0.998 g/mL, respectively).

### 2.4 Antioxidant levels

Methods for measuring levels of GSH and AsA in the lens are previously described [7]. Briefly for measuring GSH levels, the lens was homogenized in 0.1 M sodium phosphate buffer (pH 8.0) with 10% trichloroacetic acid and then centrifuged. Absorbance of the supernatant was measured at 412 nm before and 30 min after DTNB addition. For measuring AsA levels, the lens was homogenized in 0.1 M phosphate-buffered saline (pH 7.4) with metaphosphoric acid. After centrifugation, the supernatant was titrated with DCPIP. Catalase (CAT) activity was measured using a CAT assay kit (Cayman Chemical) according to the manufacturer's protocol. GSH levels in the plasma were measured using a GSSG/GSH quantification kit. AsA in the plasma was calculated from A590 absorbance using an Ascorbic Acid Colorimetric/Fluorometric Assay Kit (BioVision). Superoxide dismutase (SOD) activity was measured using an SOD assay kit-WST (Dojindo) according to the manufacturer's protocol.

### 2.5 Measurement of lens elasticity

Lens elasticity was measured using Softmeasure HG1003-SL (Horiuchi Electronics. Co., Ltd., Tokyo) [8]. The lens was removed immediately after euthanasia and placed on the mount, which was lying on the posterior pole parallel to the bottom. The tip of the height gauge was turned down to apply pressure on the lens. The pressure power and dents in the lens were measured and lens elasticity calculated using the pressure power in the lens.

### 2.6 Real-time polymerase chain reaction (PCR)

Total RNA was extracted using Sepasol-RNA I super G (Nakalai). Reverse transcription (RT) was performed using an oligo (dT)_20_ primer and 1 μg total RNA for first-standard cDNA synthesis according to commercial protocol. PCR was performed in a volume of 10 μL using the KAPA SYBR FAST qPCR kit (KAPA Biosystems, Wilmington, MA). Quantitative real-time PCR was performed using the CFX96 Real-Time system (Bio-Rad, Berkeley, CA). PCR primer sequences were as follows: mouse monoascorbate reductase 5′-CAGGGACCACCTTCCAACTC-3’ (upstream) and 5′-AACTGGATCATTGGTGGGGG-3’ (downstream): mouse dehydroascorbate reductase 5′-CACTAACAACACCAGTGCGA-3’ (upstream) and 5′-CCGCCTATGCAGTCTTTACCT-3’ (downstream): mouse glutathione reductase 5′- ACGGATGAAAAGGGCCACAT-3’ (upstream) and 5′-CTTTCCCACAGACGTCTCCC-3’ (downstream): mouse thioredoxin reductase 1 5′-TGACCAAGACATGGCCAACA (upstream) and 5′-TGTGGATTGAGCAGTCACCC-3’ (downstream).

### 2.7 Selenite injection for nuclear cataract models and G-Hsd treatment

Thirteen-day-old SD rats were randomly divided into two groups and injected with either PBS (control group) or sodium selenite (20 μmol/kg body weight). Each group was further divided into two subgroups and administered 0.2 mL PBS or an equal volume of G-Hsd (200 mg/kg body weight) via a feeding tube 4 h before the sodium selenite injection, then once a day for two days (total of three days). Four days after selenite injection, elasticity was measured in enucleated lens.

### 2.8 Statistical analysis

All *in vivo* data are reported as means ± SE. Statistical analysis of data was performed using one-way analysis of variance (ANOVA) with a post-hoc Tukey's multiple comparison test with SPSS software (version 24; IBM corporation). P values of less than 0.05 indicated statistical significance.

## 3 Results

### 3.1 Long term treatment of G-Hsd ameliorates lens hardening

**Fig. 1:**
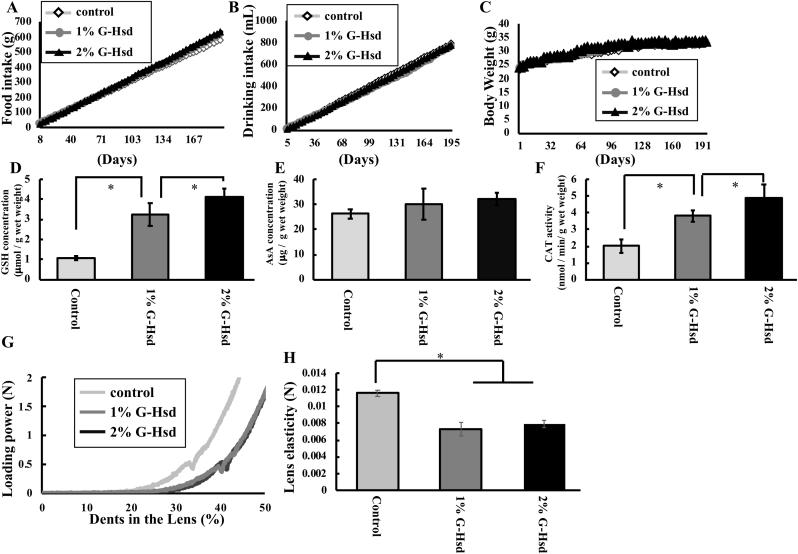
*Effect of G-Hsd consumption on antioxidant compound levels and lens hardening in C5*7BL*/6 mouse lens.* Male C57BL/6 mice were divided into three groups: treated with water, 1% (v/v) G-Hsd, or 2% (v/v) G-Hsd for 28 weeks (n = 6 mice/group). (A) Cumulative food consumption, (B) water intake, and (C) body weight are shown. (D) GSH and (E) AsA levels in the lens were measured (n = 6 lenses per group). (F) CAT activities were measured using a CAT assay kit (n = 6 lenses per group). (G) Lens hardening was measured using a texture analyser. (H) Lens elasticity was calculated as the loading power (N) to achieve 15% dents in the lens (n = 8 lenses per group). Data are presented as mean ± SEM. * indicates a significant difference versus the control group (*p* < 0.05).

Food and water intake in 1% (v/v) and 2% (v/v) G-Hsd groups was almost identical to that of the control group, and there were no significant differences in body weight among the groups (Fig. 1A–C). It is well-known that GSH and AsA levels and CAT activity decrease with age; therefore, we measured GSH and AsA levels and CAT activity in the lens of mice treated with or without G-Hsd treatment. GSH levels in the 1% and 2% G-Hsd treatment groups were significantly higher than those in the control group (Fig. 1D). Similarly, lens AsA levels increased with either 1% or 2% G-Hsd treatment (Fig. 1E). CAT activity in the lens of G-Hsd treatment groups was significantly increased compared with that in the control group in a concentration-dependent manner (Fig. 1F). Subsequently, lens hardening was measured immediately after euthanasia. The mouse lens in the control group was harder than that in 1% and 2% G-Hsd treatment groups. There was no difference between the 1% and 2% G-Hsd treatment groups (Fig. 1G). Lens elasticity was calculated as the pressure power (N) required for 15% dents in the lens, and was significantly reduced by 1% and 2% G-Hsd long-term treatment (Fig. 1H). Hardening of the lens nucleus and cortex is an important inducer of presbyopia. These results suggested that G-Hsd treatment could prevent oxidative damage in the lens and ameliorate lens hardening. Thus, G-Hsd treatment has the potential to prevent presbyopia.

### 3.2 G-Hsd treatment ameliorated the reduction of antioxidants in the plasma

**Fig. 2:**
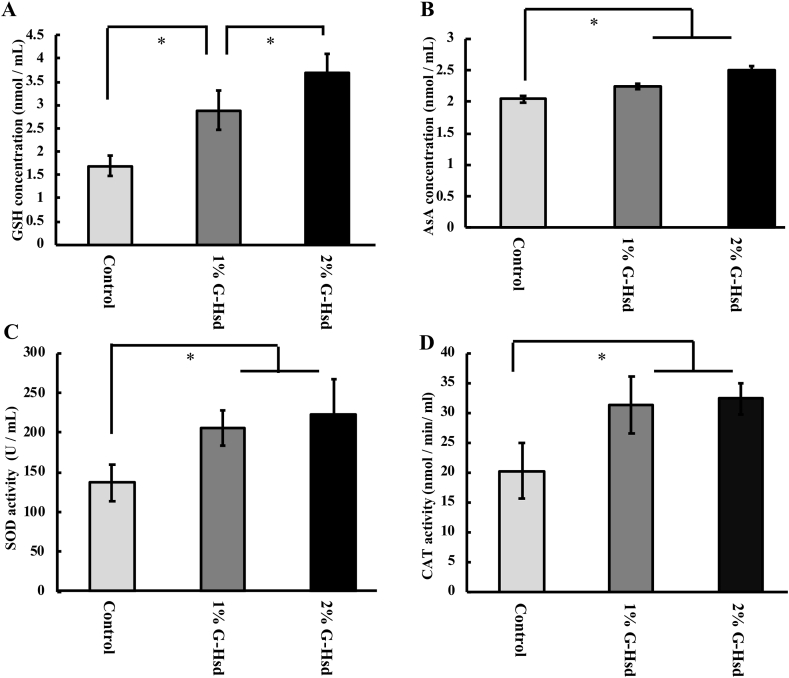
*Effect of G-Hsd consumption on plasma antioxidant levels.* Antioxidant levels in the plasma were measured after 28-week treatment of either water, 1% G-Hsd, or 2% G-Hsd. (A) GSH concentration and (B) AsA concentration were measured. (C) SOD activity in the plasma was measured using an SOD assay kit, and (D) CAT activity in the plasma was measured by a CAT assay kit (n = 3 mice per group). Data are presented as mean ± SEM. * indicates a significant difference versus the control group (*p* < 0.05).

From the above data, we hypothesized that G-Hsd could recover the redox state of the plasma, which decreases with age. We measured concentrations of antioxidants and antioxidant enzyme activities in the plasma. Administration of G-Hsd for 28 weeks significantly increased plasma GSH and AsA levels in a concentration-dependent manner (Fig. 2A and B). SOD and CAT activities were also significantly increased by G-Hsd treatment in a dose-dependent manner. These data suggest that long-term treatment with G-Hsd prevents age-related changes in redox state levels in the plasma.

### 3.3 G-Hsd treatment regulates glutathione-ascorbate cycle genes in the spleen

**Fig. 3:**
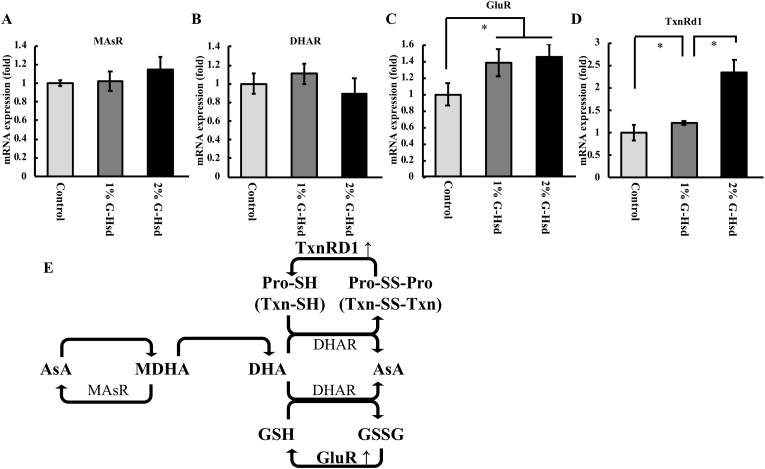
*Effect of G-Hsd consumption on mRNA expression of glutamate-ascorbate cycle-related genes in the spleen.* Mouse spleens were removed immediately after euthanasia, and the mRNA expression of (A) monoascorbate reductase (MAsR), (B) dehydroascorbate reductase (DHAR), (C) glutathione reductase (GluR), and (D) thioredoxin reductase 1 (TxnRd1) were analysed by quantitative real-time PCR. β-Actin mRNA was analysed as an internal control (n = 3 mice per group). Data are presented as mean ± SEM. * indicates a significant difference versus the control group (*p* < 0.05). (E) The glutamate-ascorbate cycle. G-Hsd affected GluR and TxnRd1 mRNA expression (red).

Next, we analysed the changes in glutathione-ascorbate cycle related gene expression within the lens following G-Hsd treatment. However, mature lens fibre cells, which compose the bulk of the lens, were unsuitable for analysis of mRNA expression due to programmed degradation of all intracellular organelles associated with mature fibre cell differentiation. Therefore, we analysed the mRNA expression levels of glutathione-ascorbate cycle-related genes in the spleen, as it is well-known that concentrations of antioxidants, such as AsA and GSH, are greater in the spleen than in plasma. This is due to the high expression of the sodium-dependent vitamin C transporters (svct), svct1 and svct2. The glutathione-ascorbate cycle in the spleen is more active than in other tissues [9]. Administration of G-Hsd increased the mRNA expression of glutathione reductase (GluR) and thioredoxin reductase 1 (TxnRd1), but not monoascorbate reductase (MAsR) and dehydroascorbate reductase (DHAR; Fig. 3A–E). These results suggest that G-Hsd consumption may increase mRNA expression levels of some genes to maintain the redox state and thereby increase antioxidant compounds in the plasma and lens.

### 3.4 G-Hsd treatment inhibits the formation of early stage nuclear cataract by selenite injection

**Fig. 4:**
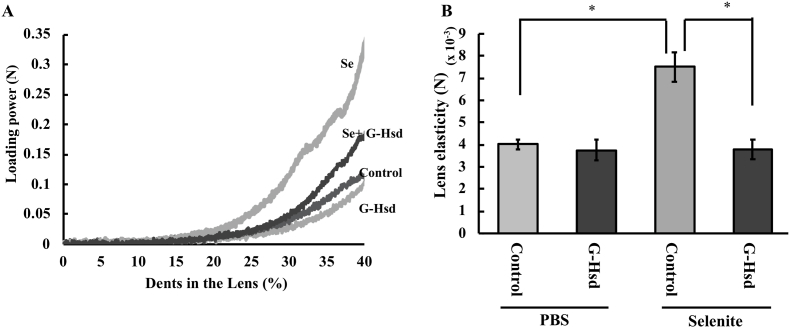
*Effect of G-Hsd consumption on lens elasticity and the formation of premature cataract upon selenite injection in rats.* Thirteen-day-old SD rats were injected with either PBS or sodium selenite and administered either pure water or G-Hsd (200 mg/kg body weight). Lens hardening was measured four days after PBS or selenite injection. (A) Lens hardening was measured using a texture analyser. (B) Lens elasticity was calculated as the loading power required to achieve 15% dents in the lens (n = 6 lenses per group). Data are presented as mean ± SEM. * indicates a significant difference versus the control group (*p* < 0.05).

It has been reported that presbyopia, which is caused by lens hardening, is an early symptom of nuclear cataract [2]. Therefore, we measured lens hardening in the selenite-induced nuclear cataract models with or without G-Hsd treatment. Thirteen-day-old SD rats were injected with sodium selenite to induce cataracts and subsequently treated with G-Hsd or pure water (control group). Lens hardening was measured four days after selenite injection. We observed no significant change in lens elasticity in the control treatment (PBS) group. Lens elasticity in the selenite injection group was significantly higher, but treatment with G-Hsd inhibited these changes (Fig. 4A and B). These results suggest that G-Hsd could prevent the onset of nuclear cataract by inhibiting lens hardening.

## 4 Discussion

The aim of the current study was to evaluate the effects of G-Hsd treatment on presbyopia development. Presbyopia is characterised by loss of elasticity of the lens and loss of near-field vision. A number of eye drops are currently under investigation in clinical trials for the treatment of presbyopia. However, the concentration of drugs reaching the lens are known to be very low as the lens is covered by the cornea and aqueous humour. Therefore, we reasoned that oral consumption of compounds that possess anti-presbyopia effects would be the optimum approach. In this study, we investigated the effects of oral administration of G-Hsd on sclerosis of the lens. Cataract causes vision loss and blindness and reduces quality of life. Cataract is also a common condition among the elderly, affecting over 90% of individuals above 80 years of age, and is the leading cause of blindness worldwide. Besides surgical removal of the cataractous lens and insertion of an artificial lens, there is no effective drug treatment to cure cataracts. It was reported that lens sclerosis and presbyopia are early observed symptoms of age-related nuclear cataract [2], and we previously reported that oral consumption of G-Hsd delayed the onset of cataract induced by sodium selenite [5]. So, we hypothesized that G-Hed could prevent lens sclerosis, and here we show that lens elasticity was maintained by G-Hsd treatment under the same experimental conditions (Fig. 4).

NF-E2-related factor 2 (NRF2) is known to modulate physiological and pathological mechanisms which in turn is influenced by oxidative stress. ROS and oxidative stress can induce NRF2 nuclear translocalization leading to the production of anti-oxidative protein such as heme oxygenase-1 (HO-1), NAD(P)H quinone dehydrogenase 1 (NQO-1), SOD and Catalase [10], and miRNA expression functioned as anti-oxidant [11,12]. It has been reported that Hst mediated activation of Nrf2 signalling pathway prevents diabetic nephropathy in rats [13], and supresses the inflammatory response induced by lipopolysaccharide (LPS) in mouse macrophage RAW 264.7 cell line [14]. For *in vivo* study, Hst treatment reduced the oxidative stress-mediated neuroinflammation, apoptotic cell death, and neurodegenerations in amyloid bata-induced Alzheimer's model animals [15]. However, there has been no reports on the effects of Nrf2 activation/translocation in the lens tissue following G-Hsd oral administration. Thus, it is important to further investigate changes in NRF2 translocalization and the activation of downstream pathways in the lens following oral consumption of G-Hsd.

We detected 1.33 ± 0.23 nmol/g Hst in the eye after 1000 mg/kg G-Hsd for 5 days administration orally. It has been reported that Hsd could permeate across the blood–brain barrier [16,17]. These data suggested that orally administered G-Hsd had direct effect for inhibit the reduction of GSH and AsA in the lens, and indirect effect for anti-presbyopia to modulate glutathione-ascorbate cycle genes in the spleen.

When the eye focuses on a distant object, the ciliary muscle is relaxed. In contrast, when the eye focuses on a near object, the ciliary muscles contract causing the ciliary body to move forward and releasing the tension on the zonules located around the lens equator. A reduction in zonule tension and ciliary muscle contraction is associated with presbyopia [3]. Takahashi and colleagues reported that periocular warming recovers accommodative ability by increasing the blood flow to the ocular region and enhancing parasympathetic responses in the ciliary muscle [18]. Similarly, blood flow to the ciliary muscle, iris, and choroid was shown to be increased after Hes or Hsd treatment [19,20]. These reports suggested that in addition to the antioxidant response demonstrated in this study, G-Hsd treatment may also increase blood flow to the ciliary muscle, iris, and choroid, thus increasing parasympathetic responses for accommodative change.

Many health supplements containing G-Hsd are associated with maintaining peripheral blood flow, blood triglyceride levels, and blood pressure. In the USA and other countries, several anti-inflammatory supplements also contain Hsd. To our knowledge, this is the first study to investigate its anti-presbyopia effects. Further studies are needed to understand the mechanisms underlying the anti-presbyopia and/or anti-cataract activity of G-Hsd before the initiation of clinical trials. G-Hsd is the first oral supplement that possesses robust anti-presbyopia and/or anti-cataract properties with the potential to prevent major eye diseases in adults.

## Authors’ contribution

YN, SE and HT defined the research theme. YN, NM, SE, MFT and HT designed the methods. YN, MA, SI, YD performed the laboratory experiments. YN, SE, NN, MFT and HT analysed and interpreted the data. YN was the major contributor in the writing of the manuscript.

## Ethics approval and consent to participate

All animal experiments were approved by the Keio University Animal Research Committee (12048-(4)).

## Funding

This work was supported by grants from the Keio Gijuku Fukuzawa Memorial Fund for the Advancement of Education, Research and from the Japan Health Foundation, and the Japan Society for the Promotion of Science KAKENHI [grants numbers 20K07184] to Y.N.

## Declaration of competing interests

The authors declare the following financial interests/personal relationships which may be considered as potential competing interests:

(1) All third-party financial support for the work in the submitted manuscript.
(2) All financial relationships with any entities that could be viewed as relevant to the general area of the submitted manuscript.
(3) All sources of revenue with relevance to the submitted work who made payments to you, or to your institution on your behalf, in the 36 months prior to submission.
(4) Any other interactions with the sponsor of outside of the submitted work should also be reported. (5) Any relevant patents or copyrights (planned, pending, or issued).
(6) Any other relationships or affiliations that may be perceived by readers to have influenced, or give the appearance of potentially influencing, what you wrote in the submitted work. As a general guideline, it is usually better to disclose a relationship than not.
